# Higher plasma levels of thymosin-α1 are associated with a lower waning of humoral response after COVID-19 vaccination: an eight months follow-up study in a nursing home

**DOI:** 10.1186/s12979-023-00334-y

**Published:** 2023-03-06

**Authors:** María del Mar Pozo-Balado, Ángel Bulnes-Ramos, Israel Olivas-Martínez, Vanesa Garrido-Rodríguez, Carmen Lozano, Ana I. Álvarez-Ríos, Berta Sánchez-Sánchez, Encarnación Sánchez-Bejarano, Isabel Maldonado-Calzado, José Manuel Martín-Lara, Juan Antonio Santamaría, Rafael Bernal, María Francisca González-Escribano, Manuel Leal, Yolanda M. Pacheco

**Affiliations:** 1 Immunology Laboratory, Institute of Biomedicine of Seville (IBiS), Immunology Service, Virgen del Rocío University Hospital (HUVR)/CSIC/University of Seville, Ave. Manuel Siurot s/n, 41013 Seville, Spain; 2 Microbiology Service, Virgen del Rocío University Hospital (HUVR), Seville, Spain; 3 Biochemistry Service, Virgen del Rocío University Hospital (HUVR), Seville, Spain; 4 Immunology Service, Virgen del Rocío University Hospital (HUVR), Seville, Spain; 5 Hogar Residencia de la Santa Caridad, Seville, Spain; 6 Internal Medicine Service, Viamed Hospital, Santa Ángela de la Cruz, Seville, Spain

**Keywords:** BNT162b2 vaccine, Magnitude and persistence of humoral response, Antibody waning, Nursing home, Thymic-function, Sj/β-TRECs ratio, RTL, Thymosin-α1, Immunesenescence, Inflammaging

## Abstract

**Background:**

Older people achieve lower levels of antibody titers than younger populations after Covid-19 vaccination and show a marked waning humoral immunity over time, likely due to the senescence of the immune system. Nevertheless, age-related predictive factors of the waning humoral immune response to the vaccine have been scarcely explored. In a cohort of residents and healthcare workers from a nursing home that had received two doses of the BNT162b2 vaccine, we measured specific anti-S antibodies one (T1), four (T4), and eight (T8) months after receiving the second dose. Thymic-related functional markers, including thymic output, relative telomere length, and plasma thymosin-α1 levels, as well as immune cellular subsets, and biochemical and inflammatory biomarkers, were determined at T1, and tested for their associations with the magnitude of the vaccine response (T1) and the durability of such response both, at the short- (T1-T4) and the long-term (T1-T8). We aimed to identify age-related factors potentially associated with the magnitude and persistence of specific anti-S immunoglobulin G (IgG)-antibodies after COVID-19 vaccination in older people.

**Results:**

Participants (100% men, *n* = 98), were subdivided into three groups: young (< 50 years-old), middle-age (50–65 years-old), and older (≥65 years-old). Older participants achieved lower antibody titers at T1 and experienced higher decreases in both the short- and long-term. In the entire cohort, while the magnitude of the initial response was mainly associated with the levels of homocysteine [β (95% CI); − 0.155 (− 0.241 to − 0.068); *p* = 0.001], the durability of such response at both, the short-term and the long-term were predicted by the levels of thymosin-α1 [− 0.168 (− 0.305 to − 0.031); *p* = 0.017, and − 0.123 (− 0.212 to − 0.034); *p* = 0.008, respectively].

**Conclusions:**

Higher plasma levels of thymosin-α1 were associated with a lower waning of anti-S IgG antibodies along the time. Our results suggest that plasma levels of thymosin-α1 could be used as a biomarker for predicting the durability of the responses after COVID-19 vaccination, possibly allowing to personalize the administration of vaccine boosters.

**Supplementary Information:**

The online version contains supplementary material available at 10.1186/s12979-023-00334-y.

## Background

Although Coronavirus disease 2019 (COVID-19) vaccines are highly effective in preventing severe disease and death, a major clinical problem remains regarding the weaker humoral response and the rapid waning in protection early after vaccination in older people [1]. Consequently, vaccinated older people still show higher mortality rates due to COVID-19 than younger populations [2, 3]. This raises a question about the optimal COVID-19 vaccination strategy among older subjects, which may differ from the general population, as it has been shown in other contexts, such as hepatitis B virus or Influenza vaccination [4–6].

There is evidence that higher antibody levels following COVID-19 vaccination are associated with better protection against severe outcomes [7–9]. In accordance, COVID-19 breakthrough infections occur mainly among individuals with lower antibody titers [10]. Therefore, understanding the factors associated with the persistence of protective antibody levels seems crucial to guide the optimal design of additional vaccine boosters. However, most studies evaluate COVID-19 vaccine responses focusing on clinical efficacy, without considering the humoral response in terms of antibody titers [2, 11], and only a few studies have addressed the problem of waning immunity by longitudinal measures of humoral responses [1].

Immunosenescence is a dysregulation of the immune system characteristic of aging linked to the involution of the thymus [12]. With aging, the thymus atrophies and declines in function, reducing the output of naïve T-cells to the periphery, which leads to clonal expansions and accumulations of exhausted/senescent memory T-cells with altered functionality [13, 14]. These alterations contribute to a chronic low-grade inflammatory state or inflammaging characteristic of advanced age [15]. Thymic involution also affects the production of thymic hormones, such as thymosin-α1, a peptide hormone with immunomodulatory properties, which induces the maturation of B and T-lymphocytes and increases the efficiency of antigen presentation, enhancing and restoring immune functions [16]. Nevertheless, the potential role of thymic function in the magnitude and long-term persistence of anti-Spike (anti-S) antibody titers along the time in COVID-19 vaccination has not yet been explored.

Our aim was to analyze potential predictive factors of the magnitude and durability of the response to COVID-19 vaccination in older people. We explored thymus-related parameters, as well as other relevant immunological factors that are also known to be altered along immunosenescence, such as the distribution of immunological subsets and levels of inflammatory and biochemical biomarkers.

## Results

### Demographical, anthropometrical and clinical characteristics of the study subjects

Ninety-eight subjects from the nursing home accepted to participate in the study, of which 88 did it from the beginning (Supplementary Fig. 1). All participants were men (100%) with a median age of 73 [64–80] years-old. Participants were categorized according to their ages, and the baseline characteristics of groups are summarized in Supplementary Table 1. The groups were: i) young (less than 50 years-old), including ten health care workers; ii) middle-age (between 50 and 65 years-old), including nine health care workers and eight residents; and iii) older (over 65 years-old), comprising 14 health care workers and 57 residents. Comorbidities were recorded from 56 out of 65 residents, being the most frequent hypertension (83.7%), diabetes (42.9%), and obesity (31.9%) (Supplementary Table 2). The comparison of thymic activity-related parameters, the distribution of immunological subsets, and soluble biochemical and inflammatory markers in the three age-based groups are summarized in Supplementary Table 3. Summing up, older participants showed a typical immunosenescent profile, characterized by lower thymic output with a trend to shortened telomeres and a generalized higher inflammatory profile compared with younger participants. They also exhibited the lowest levels of all immune subsets analyzed, except for natural killer (NK) cells that were expanded in this group.

### Age influences the magnitude and durability of the response to SARS-CoV-2 antigen

**Fig. 1:**
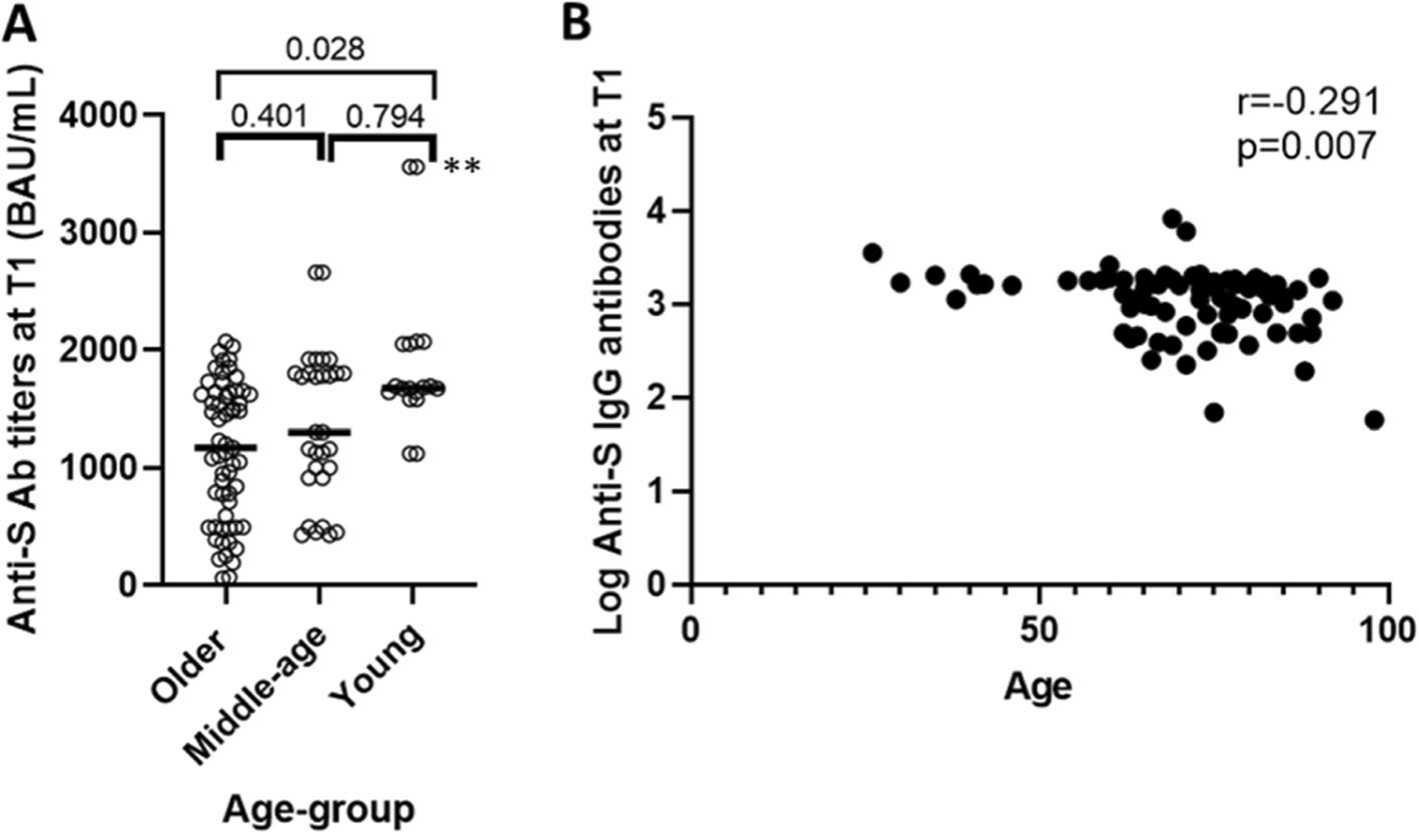
Age-related differences in the humoral response to the BNT162b2 vaccine. **A** Comparison of anti-S antibody titers between study groups at T1. Differences in anti-S IgG antibodies were maintained even when the two outliers from the young group (**) were excluded (*p* = 0.048). **B** Correlation between age and the log_10_ of anti-S antibody titers at T1. *p* values < 0.05 were considered statistically significant

The magnitude of the initial immune response achieved 1 month after the second dose of the BNT162b2 vaccine (T1) is represented in Fig. 1A. The analysis of anti-S Immunoglobulin G (IgG) antibodies at T1 revealed a strong induction of humoral immunity in response to vaccination in all study participants, with a 100% of seroconversion rate in the entire cohort, although we found that anti-S antibody titers achieved at T1 were significantly lower in older adults compared to younger participants. However, it is remarkable that participants of the older group showed a mixed phenotype since a substantial number of participants of the older group had T1 responses as good as the young group. Interestingly, these two subgroups of older participants differed in thymosin-α1 levels as well as in other studied variables (Supplementary Fig. 2). Moreover, we observed a negative correlation between the magnitude of the initial response and age (*r* = − 0.291; *p* = 0.007) (Fig. 1B).

**Fig. 2:**
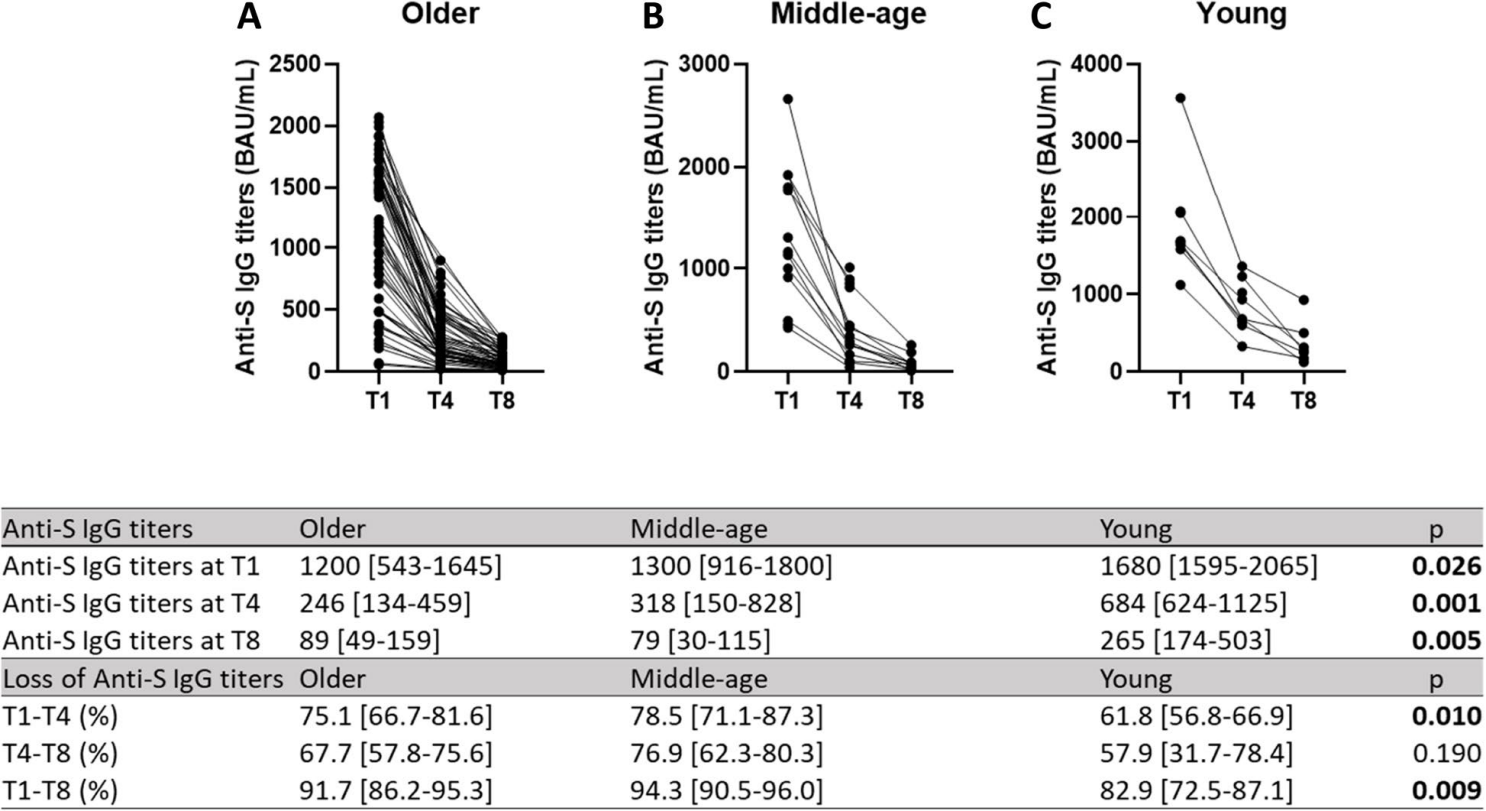
Longitudinal dynamics of Anti-S antibody titers by age-groups. Follow-up of anti-S antibody titers between T1 (one month after the second dose), T4 (four months after the second dose) and T8 (eight months after the second dose) in **A**) Young, **B**) Middle-Age and **C**) Older groups. For all comparisons, *p* values 0.05 were considered statistically significant. Friedman test *p* < 0.001 for all comparisons

The dynamics of anti-S antibodies over time are represented in Fig. 2. In the entire population, a decline in antibody titers over time was observed (Friedman test *p* < 0.001). Anti-S IgG titers rapidly decreased for the first 3 months (74% between T1 and T4), followed by a relatively slow decrease thereafter (92.0% between T1 and T8). By groups, median anti-S IgG titers decreased between T1 and T4 by approximately 2.6-folds in young participants, compared with a 4.7 and 4-folds decrease in participants of the middle-age and older groups (*p* = 0.010). In the entire population, the rate of seropositivity also declined from 100% at T1 to 95.5% at T4, and 88.8% at T8. At T4, four participants from the old-group switched to seronegative, whereas at T8, two participants from the middle-age-group and 12 from the old-group became seronegative. No correlation was found between the loss of anti-S IgG titers and the age of participants, neither in the short-term nor in long-term.

Interestingly, we observed that anti-S antibody titers achieved at T1, T4, and T8 strongly correlated with each other (Supplementary Fig. 3). No correlations between the anti-S antibody titers achieved at T1 and the loss of anti-S antibody levels at the short-term (*r* = − 0.163, *p* = 0.159) or the long-term (*r* = 0.017, *p* = 0.891) were observed.

### Age-related factors associated to the magnitude of the initial response to two doses of BNT162b2 vaccine

**Fig. 3:**
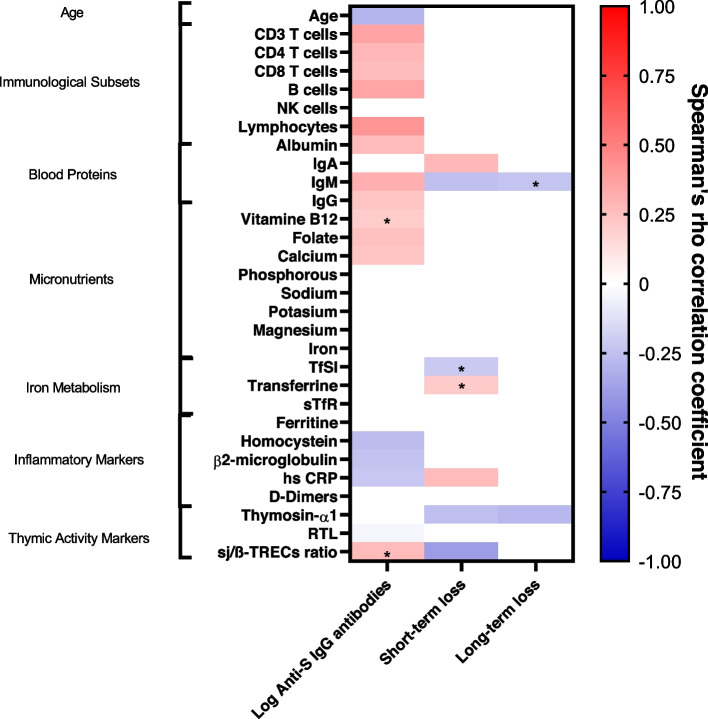
Factors associated with the magnitude and loss of anti-S titers after BNT162b2 vaccination. Spearman’s correlation analysis between potential age-related factors associated to the magnitude of the initial response to the vaccine (T1, one month after the second dose of the vaccine), as well as to the short-term loss (between T1 and T4) and to the long-term loss (between T1 and T8). Color intensity of boxes represents Spearman’s rank correlation coefficient value as indicated in the color legend. All colored boxes represent statistically significant correlations (*p* values < 0.05). * *p* values between 0.1 and 0.05

To address the potential effects of thymic activity-related biomarkers, the distribution of the immune cell subsets, biochemical and inflammatory markers on the magnitude of the initial response to the vaccine, we analyzed the potential relationships between these parameters and the anti-S titers achieved at T1 (Fig. 3). Regarding the potential role of the thymic activity parameters in the initial response to the vaccine, we only found a trend to a positive correlation between the ratio of signal-joint to beta T-cell receptor excision circles (sj/β-TRECs) and anti-S IgG titers at T1. Interestingly, although the levels of thymosin-α1 at T1 did not correlate with anti-S IgG titers at T1, they did at T8 (*r* = 0.334, *p* = 0.01). The magnitude of the response also correlated positively to total lymphocytes, CD3, CD4, CD8 T, and B cell counts, but was not associated with NK cells. Attending to the biochemical markers analyzed, we observed that, except for immunoglobulin A (IgA), the anti-S IgG titers achieved at T1 were positively associated with the level of all blood proteins analyzed, as well as with the levels of vitamin B12, folate, and calcium. Examining the potential effect of inflammatory markers, we found that homocysteine, β2-microglobulin, and high sensitivity C reactive protein (hsCRP) were negatively associated with the magnitude of the initial response to the vaccine. As can be seen in Supplementary Fig. 4, all these correlations depended on the study group.

We next performed a multivariable analysis in which all variables that significantly correlated in the bivariate analysis with the anti-S antibody titers achieved at T1, were included. Linear multivariable analysis showed that homocysteine was the unique variable independently associated with the magnitude of the initial response to the vaccine [β coefficient; (95% confidence interval (CI), − 0.155 (− 0.241 to − 0.068); *p* = 0.001] **(**Table 1**)**.

**Table 1:** Linear regression multivariable analyses of the magnitude of the initial response to the vaccine one month after the second dose of the BNT162B2 vaccine (T1) and loss of anti-S IgG titers at the short- (T1-T4) and long-term (T1-T8)

Parameter	β coefficient (95% CI)	***p***
**Anti-S antibody titers (T1)**		
Age (years)	−0.512 (− 0.021 to 0.001)	0.100
Albumin (g/dL)	0.134 (− 0.197 to 0.434)	0.455
Total IgM (mg/dL)	0,1185 (−0.001 to 0.002)	0.599
Total IgG (mg/dL)	0.189 (0.000 to 0.001)	0.295
Folate (ng/mL)	−0.068 (− 0.483 to 0.365)	*0.062*
Calcium (mg/dL)	0.030 (−0.213 to 0.256)	0.855
Homocysteine (mg/L)	−0.155 (− 0.241 to − 0.068)	**0.001**
β2-microglobulin (mg/L)	0.264 (− 0.012 to 0.162)	*0.089*
hsCRP (mg/L)	− 0.085 (− 0.026 to 0.013)	0.502
CD3 (cells/μL)	− 0.039 (0.000 to 0.000)	0.753
B cells (cells/μL)	0.142 (0.000 to 0.002)	0.257
**Short-term loss of anti-S antibody titers (T1-T4)**		
Total IgA (mg/dL)	0.010 (−0.012 to 0.031)	0.373
Total IgM (mg/dL)	−0.020 (− 0.055 to 0.015)	0.253
hsCRP (mg/L)	0.440 (−0.368 to 1.248)	0.280
Thymosin-α1 (ng/mL)	−0.168 (− 0.305 to − 0.031)	**0.017**
sj/β-TRECs ratio	− 0.558 (−1.118 to 0.002)	*0.074*
**Long-term loss of anti-S antibody titers (T1-T8)**		
Total IgM (mg/dL)	−0.017 (− 0.037 to 0.003)	*0.089*
Thymosin-α1 (ng/mL)	− 0.123 (− 0.212 to − 0.034)	**0.008**

*T1* one month after the second dose, *T4* four months after the second dose, *T8* eight months after the second dose, *CI* Confidence interval, *hsCRP* high sensitivity C-reactive protein

### Age-related factors as predictors of the loss of anti-S antibody titers along the time

We analyzed the relationships between the percentages of loss of anti-S antibody titers at both, the short- (T1-T4) and the long-term (T1-T8), and thymus activity-related parameters, the distribution of immune cell subsets, levels of biochemical markers and inflammatory-related biomarkers (Fig. 3). Attending to thymus activity-related parameters in the entire population, we observed a negative correlation between the sj/β-TRECs ratio and the short-term loss of antibodies. Interestingly, the levels of thymosin-α1 were also inversely correlated with the short-term loss of antibodies (*r* = − 0.251, *p* = 0.046). This negative correlation was maintained in the long-term (*r* = − 0.279, *p* = 0.034). Regarding the biochemical markers analyzed, we found that the short-term loss of anti-S antibodies correlated positively with total IgA, but was negatively associated with total immunoglobulin M (IgM). We also observed that both transferrin and transferrin saturation index (TfSI) tended to correlate with the short-term loss of anti-S titers. Respect the inflammatory status, only hsCRP levels were associated with the short-term loss of anti-S titers, but this correlation was not maintained in the long-term.

We next performed a multivariable analysis to determine independent factors affecting the loss of anti-S IgG titers in both the short- and long-term. We included all variables significantly associated in the bivariate analysis with the short-term loss of antibodies. For the long-term multivariate analysis, in addition to thymosin-α1, which was the unique variable that significantly correlated with the long-term loss of antibodies in the bivariate analysis, total IgM was also included due to its high tendency to correlate (*r* = − 0.235, *p* = 0.055). Linear regression analysis revealed that thymosin-α1 at T1 was the unique variable independently associated with the loss of anti-S antibody titers at both, the short-term [β coefficient − 0.168; 95% confidence interval [CI], (− 0.305 to − 0.031); *p* = 0.017] and at long-term [β coefficient − 0.123; 95% confidence interval [CI], (− 0.212 to − 0.034); *p* = 0.008] (Table 1).

## Discussion

In this study, carried out in a nursing home, the humoral immunogenicity elicited by the BNT162b2 vaccine and the durability of such response along the time were conditioned by age and age-related factors. While the magnitude of the initial response to the vaccine was independently associated with the inflammatory status of the participants, the durability of such response in the long-term (up to 8 months), was only predicted by the previous levels of thymosin-α1, a thymic-derived immunomodulatory hormone.

Immunity against emerging viruses and de novo vaccine responses depend on the efficacy of mounting primary responses, which is known to be impaired in older adults, mainly due to immunosenescence [12]. Thus, the benefits of vaccination in the prevention of infectious diseases are limited in older people, who normally show defective humoral responses to several vaccines [4], such as Influenza [5, 6], Hepatitis B virus [17], and SARS-CoV2 [1, 18–21]. In fact, despite the considerable high efficacy of the available SARS-CoV-2 mRNA vaccines, aged people, even vaccinated, maintain increased mortality rates due to COVID-19 than younger subjects [22].

Preclinical studies in animal models, as well as vaccine efficacy studies and the early use of convalescent plasma, suggest that neutralizing antibodies are the best correlators of protection against the more severe outcomes of COVID-19 [1, 4, 8, 9, 23–26]. In our study, although the seroconversion rate was 100% for all vaccinees, the magnitude of the initial humoral response was significantly lower in the oldest participants, which was in accordance with previous reports [1, 18]. We also observed a negative association between anti-S IgG titers 1 month after vaccination with the age of the participants, a correlation that was maintained 4 months later (data not shown). In addition to the diminished magnitude of the initial response among older adults, current evidence indicates the increasing risk of SARS-CoV-2 symptomatic infection with time due to the waning of humoral immunity [1, 10]. We found that anti-S IgG titers rapidly decayed early after vaccination in all participants without exception, but especially among aged vaccinees, in accordance with previous reports [19]. On the other hand, the loss of anti-S IgG titers did not correlate with the age of the participants. Consequently, the identification of reliable age-related factors associated with the immunogenicity and durability of the response over time is mandatory and could help to improve personalized vaccination/boosting strategies.

It is known that the distribution of immune cell subsets affects the immune responses against vaccine antigens [27–29]. Moreover, age-dependent differences in T and B lymphocytes, as well as in NK populations distribution and functions have been well-defined [30]. Thus, their relationship with antibody responses following vaccination and the durability of the humoral response over time could provide valuable information for understanding protective long-term immunity against COVID-19. As expected, in our cohort, the distribution of the peripheral immune cell subset depended on age. Thus, older participants showed lower levels of total lymphocytes, CD3, CD4, CD8, and B lymphocytes, but higher levels of NK cells, an immune profile tightly related to immunosenescence [30–33]. Interestingly, all immune subsets analyzed correlated positively to the magnitude of the humoral response to the vaccine, except NK cells, which were not associated with such a response.

Another age-related immune defect is inflammaging, a low grade chronic persistent inflammatory phenotype found in older populations [34], that contributes to the decline in the immune response and has been shown to inhibit immune responses to vaccination [17, 35, 36]. Accordingly, older participants of our study showed significantly higher levels of inflammatory markers, including homocysteine, β2-microglobulin, and D-dimers, and a trend to higher levels of hsCRP. All of these markers correlated negatively with the magnitude of the response, but only the levels of hsCRP correlated with the short-term loss of anti-S antibody titers. These results point out that pharmacological strategies aiming at blocking baseline inflammation can be potentially used to boost COVID-19 vaccine responses, but would not be probably enough to avoid the intense waning of humoral protection.

The aging of the immune system is proposed to impact COVID-19 vaccination efficiency [37]. One of the main factors underlying immunosenescence is age-related thymic involution [15]. Hence, we hypothesized that deficient thymic function related to aging might impact COVID-19 vaccination efficiency and should also be considered a key player in aged populations. In our study, older participants showed a reduced thymic output, determined by a lower sj/β-TRECs ratio in the periphery. Interestingly, the RTL of immune cells from the older group tended to be shorter than that from the other groups, suggesting an age-dependent shift from naïve to memory phenotype induced by homeostatic T-cell proliferation to compensate for the diminished T-cell thymic output in older participants. Interestingly, the sj/β-TRECs ratio tended to correlate positively with the magnitude of the response to the vaccine, also being associated with the short-term loss of anti-S IgG titers. In accordance, diminished responsiveness to vaccination against other viruses as yellow fever virus has been previously associated with lower thymus activity [38]. Remarkably, thymus activity does not rely only on cellular output, but also on its production of immunomodulatory hormones. We found that although the levels of thymosin-α1 at T1 did not correlate with anti-S IgG titers at T1, they did at T8. Moreover, and despite there being no differences in the levels of thymosin-α1 between age groups, we found that older people with a higher magnitude of response at T1 had higher levels of thymosin- α1. Furthermore, levels of thymosin- α1 predicted the short- and long-term loss of anti-S IgG titers, being the unique variable independently associated in the multivariable analysis with the loss of antibodies at both the short- and the long-term. Our results are of relevance since thymosin-α1, a peptide with immunomodulatory properties that induces differentiation of B and T lymphocytes has already been used in several clinical trials (phase I/II/III/IV) and research settings, such as different types of cancer, sepsis, and infectious diseases, including COVID-19 [39–44]. Notably, its use as an adjuvant to the Influenza and hepatitis B vaccines improved the immunogenicity and durability of the humoral responses, showing to be safe and well tolerated [39, 40, 42]. Future research addressing the potential benefit of using this immunomodulator as an adjuvant of COVID-19 vaccination would be desirable since it could also improve the durability of responses. Also remarkable, the potential role of the thymus in the severity of COVID-19 has been discussed [45–49], and thymosin-α1 has also been used as a treatment for severe COVID-19, although with controversial results [44, 50, 51].

Our study has several limitations. We present an exploratory study with a relatively small sample size but fully representative of a nursing home, allowing us a close monitor follow-up. On the other hand, all participants from the study were men, and consequently, our results might not be extrapolated to cohorts including women. However, this approximation allowed us to avoid the potential influence of gender in age-related factors measured and its relation with the magnitude and loss of antibodies over time. Finally, the vaccination of residents of nursing homes was considered a priority, and the urgent vaccination of this especially vulnerable population did not allow us to collect samples before vaccinations. Nevertheless, our results support and extend previous observations from COVID-19 vaccine responses following vaccination in older people and raise novel potential approaches to optimize COVID vaccination/boosting protocols in this vulnerable population.

## Conclusion

In conclusion, while the magnitude of the initial response to the vaccine was associated with the inflammatory status of the participants, the durability of such response in the short- and long-term was predicted by the previous levels of thymosin-α1. Our results suggest that thymosin-α1 could be used as a biomarker for the durability of the responses after COVID-19 vaccination, possibly allowing to personalize the administration of vaccine boosters.

## Methods

### Study design and participants

This study was performed at the “Santa Caridad elderly home” (Seville, Spain), a nursing home where only men are admitted as residents. To avoid gender differences due to the different distribution of sexes along age, only men health workers were included in this study. Residents and health workers, neither previously diagnosed with COVID-19 (determined by medical history) nor under immunosuppressive therapy that had received two doses of the BNT162b2 vaccine, were invited to participate in the study. Participants who acquired SAR-COV-2 infection during the follow-up were not considered thereafter. A schematic representation of the vaccine administration protocol and sample collection is shown in Supplementary Fig. 1. Participants received the first and second doses of the BNT162b2 vaccine on January 4th and 25th 2021 respectively. Blood samples started to be collected one-month after the second dose (T1) to assess factors potentially associated with the magnitude of the initial response and also four (T4) and eight (T8) months later to evaluate the potential predictive factors of the durability of such response at both, short-term (T1-T4) and long-term (T1-T8). Specific antibody titers were determined at all time-points, whereas the rest of the parameters were analyzed only at T1. The study was performed according to the Helsinki Declaration of the World Medical Association and approved by the Research Ethics Committee of the Virgen del Rocío and Virgen Macarena University Hospitals (PEIBA Acta CEI_02/2021). Written informed consent was obtained from all participants.

### Quantification of IgG anti-SARS-CoV-2 antibodies

IgG antibodies against the trimeric SARS-CoV-2 Spike protein were quantified in serum samples by using chemiluminescence assay (LIAISON® SARS-CoV-2 TrimericS IgG, Diasorin S.p.A, Saluggia, Italy) and run on a DiaSorin LIAISON XL platform (DiaSorin, Stillwater, USA). According to the manufacturer’s data, the sensitivity and specificity of this test were 98.7 and 99.5%, showing a good correlation with the microneutralization test (Positive percent agreement (PPA): 100%, Negative percent agreement (NPA): 96.9%) [22]. Antibody concentration, expressed as Binding Arbitrary Units (BAU)/mL, was automatically calculated by the analyzer from Arbitrary Units (AU/mL) by the following conversion formula: AU/mL*2.6 = BAU/mL, and a positive result was considered as ≥33.8 BAU/mL.

### Immune subsets

Absolute numbers of lymphocytes, CD3, CD4, and CD8 T-cells, B-cells and NK-cells were routinely determined in fresh blood samples collected at T1, with a Navious EX flow cytometer (Beckman-Coulter, Brea, California), by standard procedures at the Immunology Service of our hospital.

### Biochemical soluble markers

Biochemical soluble markers, including blood proteins, micronutrients, soluble iron metabolism-related markers, and inflammatory biomarkers, were routinely determined by standard procedures at the Biochemistry Service of our hospital. Briefly, total iron, transferrin, and soluble transferrin receptor (sTfR) were measured by photometry and ferritin by particle enhanced immunoturbidimetric assay in a Hitachi Cobas C702 modular analyzer (Roche Diagnostics, Rotkreuz, Switzerland). hsCRP and β2-microglobulin levels were determined in frozen serum samples with an immunoturbidimetric assay using Cobas 701 (Roche Diagnostics, Mannheim, Germany). Measurements of the levels of homocysteine were performed by photometry according to the manufacturer’s instructions, and D-dimer levels were quantified by using an automated latex enhanced immunoassay using frozen plasma samples (HemosIL D-Dimer HS 500, Instrumentation Laboratory, Bedford, Massachusetts).

### Thymic function-related determinations

#### DNA extraction

DNA was extracted from cryopreserved peripheral blood mononuclear cells (PBMCs) by using Omega BIO-TEK, E.Z.N.A blood DNA Mini Kit. Extracted DNA was used for both, quantification of the relative telomere length (RTL) and the thymic output (sj/β-TRECs ratio).

#### RTL quantification

For RTL quantification, copy number quantifications were performed by quantitative polymerase chain reaction (qPCR). We determined the ratio between the number of copies of the telomere sequence and the single copy gene Beta-globin. The fluorescent reading for the copy number quantification was performed in a Light-cycler 480 (Roche).

#### Thymic output quantification (sj/β-TRECs ratio)

The thymic output was calculated as the sj/β-TRECs ratio by droplet digital PCR (ddPCR) in a single reaction using a QX200 system (BIORAD). The results were analyzed by using the Quantasoft 1.7.1 Software.

#### Thymosin-α1 quantification

Thymosin-α1 was quantified in plasma samples using the Human Thymosin-α1 competitive ELISA kit (MyBiosurce®) following the manufacturer’s instructions.

More detail on the methods can be found in Supplementary Methodology.

### Statistical analysis

Continuous variables were recorded as median values and interquartile ranges [IQR], and categorical variables were recorded as numbers and percentages n (%). For continuous variables, multiple comparisons between groups were made using the non-parametric Kruskal-Wallis test, and comparisons between two groups were performed using the Mann-Whitney U-test. The Friedman test and Bonferroni adjustment to a series of post hoc Wilcoxon matched pairs tests were applied for multiple longitudinal comparisons. Comparisons between categorical variables were performed by using the χ^2^ or the Fisher exact test as appropriate. Correlations between quantitative variables were explored by using the Spearman correlation coefficient test. Linear regression multivariable analyses were performed to determine predicting factors of the magnitude of the initial response and loss of antibody titers at both short- and long-term. Except for the Bonferroni corrections (*p* < 0.017), *p* values *p* < 0.05 were considered statistically significant. Statistical analyses were performed using the SPSS software (SPSS 21.0, Chicago, IL), and the graphics were generated with the Prism program (GraphPad Software v8.0).

## Supplementary Information

**Additional file 1: Supplementary Fig. 1.** Study design and Flow-chart.

**Additional file 2: Supplementary Table 1.** Demographical and anthropometrics characteristics of the study population.

**Additional file 3: Supplementary Table 2.** Comorbidities and behavioral factors of residents from the Santa Caridad Nursing Home.

**Additional file 4: Supplementary Table 3.**Thymic activity, Biochemical, Inflammatory and Immunological profiles of the study populations one month after the second dose (T1).

**Additional file 5: Supplementary Fig. 2.** Levels of Thymosin-α1 in higher vs. lower responders of the older group.

**Additional file 6: Supplementary Fig. 3.** Correlations among Log of anti-S antibody titers at all different study points.

**Additional file 7: Supplementary Fig. 4.** Factors associated with the magnitude of the initial response to the BNT162B2 vaccine at T1 by age-groups.

**Additional file 8.** Detailed methodology.

## Data Availability

The datasets used and/or analyzed during the current study are available from the corresponding author upon reasonable request.

## Abbreviations

Anti-S: Anti-Spike
AU: Arbitrary units
BAU: Binding arbitrary units
COVID-19: Coronavirus disease 2019
ddPCR: Droplet digital polymerase chain reaction
hsCRP: High sensitivity C reactive protein
IgA: Immunoglobulin A
IgG: Immunoglobulin G
IgM: Immunoglobulin M
NK: Natural killer
NPA: Negative percent agreement
PBMCs: Peripheral blood mononuclear cells
PPA: Positive percent agreement
qPCR: Quantitative polymerase chain reaction
RTL: Relative telomere length
sj/β-TRECs ratio: Ratio of signal-joint to beta T-cell receptor excision circles
sTfR: Soluble transferrin receptor
TfSI: Transferrin saturation index
