# Supplementary material for: Higher plasma levels of thymosin-α1 are associated with a lower waning of humoral response after COVID-19 vaccination: an eight months follow-up study in a nursing home
Source: Immun Ageing. 2023 Mar 6;20:9. doi: 10.1186/s12979-023-00334-y (PMC9986663; doi:10.1186/s12979-023-00334-y)

# Additional information 1.

## Supplementary Figure 1. Study design and Flow-chart.

(S1A) Schematic representation of the administration of the BNT162b2 vaccine and time points of study. Specific antibody titers were determined at all time-points whereas the rest of the parameters were only analyzed at T1. (S1B) Flowchart for participants. Our study cohort included a dynamic population, with different inputs and outputs during the follow-up period. Ninety-eight subjects from the elderly home accepted to participate in the study, of which 88 did it from the beginning (T1). Eight people entered the study at T4. Two residents died between T1 and T4 (by causes not related to the administration of the vaccine) and 6 participants with loss of follow-up (LFU) were definitively excluded from the study, whereas 2 participants had no available samples (N/A) at this time-point. Two new subjects entered the study at T8; two residents died between T4 and T8 (by causes not related to the vaccine) and two residents with a confirmed PCR for SARS-CoV-2 diagnosis between T4 and T8, were also excluded from the study.


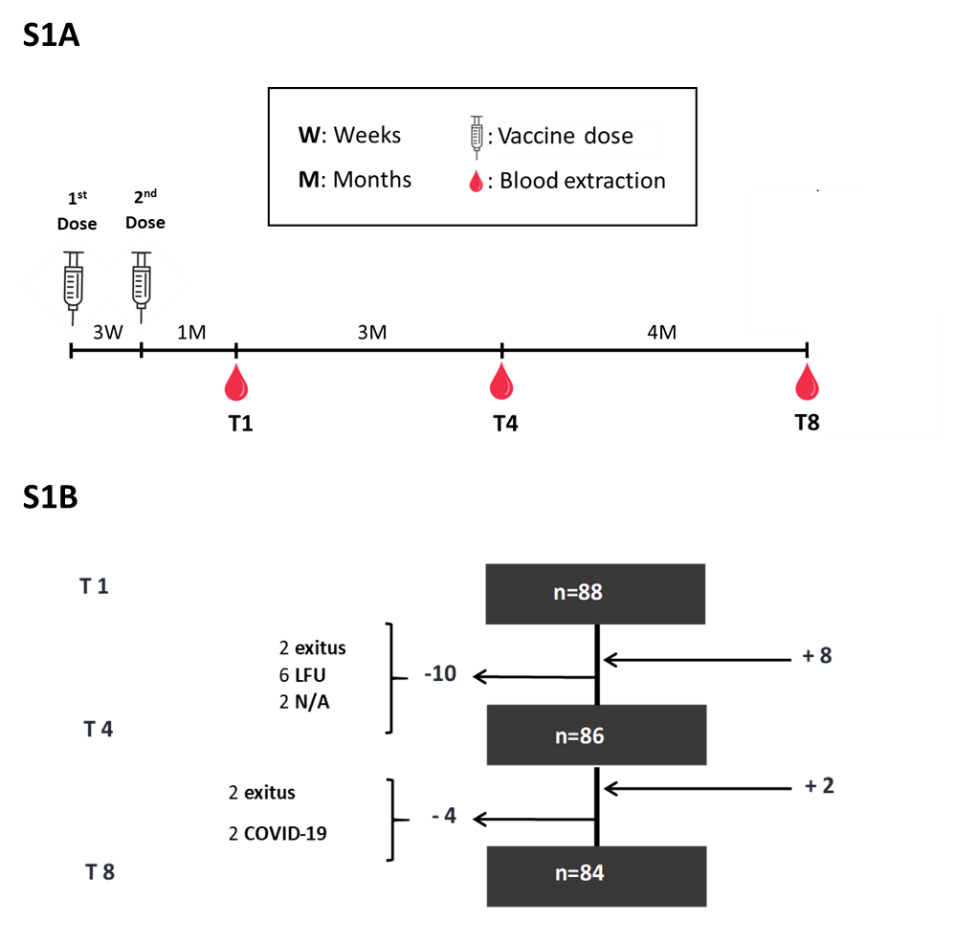

Supplement: Supplementary file 1 — Additional file 1: Supplementary Fig. 1. Study design and Flow-chart. [file 12979_2023_334_MOESM1_ESM.docx]
