# Supplementary material for: Higher plasma levels of thymosin-α1 are associated with a lower waning of humoral response after COVID-19 vaccination: an eight months follow-up study in a nursing home
Source: Immun Ageing. 2023 Mar 6;20:9. doi: 10.1186/s12979-023-00334-y (PMC9986663; doi:10.1186/s12979-023-00334-y)
Supplement: Supplementary file 2 — Additional file 2: Supplementary Table 1. Demographical and anthropometrics characteristics of the study population. [file 12979_2023_334_MOESM2_ESM.docx]

# Additional information 2

## Supplementary Table 1. Demographical and anthropometrics characteristics of the study population.

| **Characteristic** | **Younger (n=10)** | **Middle-Age (n=17)** | **Older (n=71)** | ***p*** |
| --- | --- | --- | --- | --- |
| Age, years | 39 [31-41] | 62 [59-64] | 77 [71-82] | **<0.001** |
| Gender, men n (%) | 10 (100) | 17 (100) | 71 (100) | 1.000 |
| BMI (Kg/m2) | 24.6 [23.5-29.6] | 25.8 [23.6-29.7] | 26.4 [24.8-30.4] | 0.448 |

Continuous variables are expressed as median [IQR] and categorical variables as n (%). Comparisons between continuous variables were made by using the non-parametric Kruskal-Wallis test, while the Chi-square test was used for comparison between categorical variables. *p* values <.05 were considered statistically significant. BMI: Body Mass Index.
