# Supplementary material for: Higher plasma levels of thymosin-α1 are associated with a lower waning of humoral response after COVID-19 vaccination: an eight months follow-up study in a nursing home
Source: Immun Ageing. 2023 Mar 6;20:9. doi: 10.1186/s12979-023-00334-y (PMC9986663; doi:10.1186/s12979-023-00334-y)
Supplement: Supplementary file 3 — Additional file 3: Supplementary Table 2. Comorbidities and behavioral factors of residents from the Santa Caridad Nursing Home. [file 12979_2023_334_MOESM3_ESM.docx]

**ADDITIONAL INFORMATION 3**

## Supplementary Table 2. Comorbidities and behavioral factors of residents from the Santa Caridad NURSING Home.

| **Comorbidities n (%)** | **Young** | **Middle-Age (n=7)** | **Older (n=49)** |
| --- | --- | --- | --- |
| **Cancer** | --- | 1 (14.3) | 7 (14.3) |
| **Overweight*** | --- | 1 (14.3) | 16 (34) |
| **Obesity*** | --- | 2 (28.3) | 15 (31.9) |
| **Renal Chronic Disease** | --- | 1 (14.3) | 7 (14.3) |
| **COPD** | --- | 2 (28.3) | 10 (20.4) |
| **Iskemic Heart Disease** | --- | 2 (28.6) | 8 (16.3) |
| **Diabetes** | --- | 5 (71.4) | 21 (42.9) |
| **Cerebrovascular Disease** | --- | 1 (14.3) | 10 (20.4) |
| **Arterial Hypertension** | --- | 3 (42.9) | 41 (83.7) |
| **≥ 5 Comorbidities** | --- | 3 (42.9) | 18 (36.7) |
| **Behavioral factors n (%)** |  |  |  |
| **Smoker** | --- | 4 (57.1) | 26 (53.1) |
| **Drinker** | --- | 4 (57.1) | 21 (42.9) |

Comorbidity data was available from 56 residents out of the entire group of residents (n=65). Variables are represented as n (%). COPD: Chronic Obstructive Pulmonary Disease. * N=47 in the older group.
