# Supplementary material for: Higher plasma levels of thymosin-α1 are associated with a lower waning of humoral response after COVID-19 vaccination: an eight months follow-up study in a nursing home
Source: Immun Ageing. 2023 Mar 6;20:9. doi: 10.1186/s12979-023-00334-y (PMC9986663; doi:10.1186/s12979-023-00334-y)
Supplement: Supplementary file 4 — Additional file 4: Supplementary Table 3.Thymic activity, Biochemical, Inflammatory and Immunological profiles of the study populations one month after the second dose (T1). [file 12979_2023_334_MOESM4_ESM.docx]

**ADDITIONAL INFORMATION 4.**

## Supplementary Table 3. thYMIC ACTIVITY, BIOCHEMICAL, INFLAMMATORY AND Immunological profiles of the study populations one month after the second dose (T1).

| **Thymus-activity parameter** | | **Older (n=71)** | **Middle-Age (n=17)** | **Young (n=10)** | ***p*** |
| --- | --- | --- | --- | --- | --- |
| RTL | 1361 [613-2513] | | 2402 [805-3535] | 953 [771-3863] | 0.120 |
| sj/β TRECs ratio | 2.7 [1.2-5.3] | | 9.6 [1.6-15.2] | 13.8 [6.8-17.7] | **0.017** |
| Thymosin-α1 (ng/mL) | 41 [28.9-58.2] | | 34.9 [25.0-40.5] | 48.1 [35.2-67.5] | 0.266 |
| **Biochemical Markers** |  | |  |  |  |
| Albumin (g/dL) | 4.8 [4.6-5.1] | | 4.9 [4.6-5.1] | 5.1 [5-5.2] | 0.132 |
| IgA (mg/dL) | 297 [213-459] | | 283 [220-405] | 234 [187-308] | 0.505 |
| IgM (mg/dL) | 73 [41-118] | | 96 [55-158] | 112 [102-136] | 0.158 |
| IgG (mg/dL) | 1221 [1028-1339] | | 1130 [1048-1324] | 1146 [1046 [1267] | 0.770 |
| Vitamin B12 (pg/mL) | 334 [239-463] | | 342 [269-473] | 468 [306-527] | 0.304 |
| Folate (ng/mL) | 5 [3.5-7.8] | | 6.7 [4.5-8.7] | 6.1 [5.3-7.2]] | 0.155 |
| Calcium (mg/dL) | 10.2 [9.9-10.5] | | 10.1 [9.9-10.3] | 10.2 [10.1-10.3] | 0.786 |
| Phosphorous (mg/dL) | 3.2 [3.0-3.5] | | 3.4 [3.0-3.9] | 3.6 [3.0-3.8] | 0.180 |
| Sodium (mEq/L) | 151 [148-155] | | 150 [146-153] | 148 [146-150] | 0.141 |
| Potassium (mEq/L) | 4.8 [4.5-5.3] | | 4.8 [4.7-5.5] | 4.6 [4.4-5.0] | 0.300 |
| Magnesium (mg/dL) | 2.2 [2.1-2.4] | | 2.2 [2.1-2.4] | 2.2 [2.0-2.3] | 0.752 |
| Iron (µg/dL) | 91 [62-120] | | 86 [50-120] | 109 [96-126] | 0.345 |
| TfSI (%) | 26 [17-33] | | 21 [11-30] | 29 [26-41] | 0.189 |
| Transferrin (mg/dL) | 281 [253-315] | | 320 [255-366] | 286 [265-328] | 0.207 |
| sTfR (mg/L) | 3.6 [2.9-4.6] | | 3.2 [2.9-4.7] | 2.7 [2.4-4.4] | 0.202 |
| **Inflammatory Markers** |  | |  |  |  |
| Ferritin (ng/mL) | 88 [38-180] | | 71 [26-187] | 148 [92-198] | 0.579 |
| Homocysteine (mg/L) | 2.6 [2.1-3.6] | | 2.4 [1.9-2.7] | 1.7 [1.6-2.0] | **0.001** |
| β-2 microglobulin (mg/L) | 2.8 [2.2-3.8] | | 2.4 [2.0-3.6] | 1.7 [1.4-1.8] | **<0.001** |
| hsCRP (mg/L) | 2.1 [1.1-4.9] | | 2.1 [0.8-8.1] | 1.2 [0.6-2.0] | 0.119 |
| D-dimers (ng/mL) | 570 [380-1030] | | 375 [322-665] | 210 [170-300] | **<0.001** |
| **Immune cellular subsets** |  | |  |  |  |
| CD3 (cells/µL) | 965 [814-1257] | | 1331 [1144-1612] | 1440 [996-1629] | **0.010** |
| CD4 (cells/µL) | 652 [517-855] | | 912 [647-978] | 888 [637-1045] | **0.041** |
| CD8 (cells/µL) | 291 [212-435] | | 472 [253-678] | 521 [349-635] | **0.043** |
| B cells (cells/µL) | 102 [61-134] | | 164 [91-259] | 176 [131-255] | **0.006** |
| Nk cells (cells/µL) | 317 [216-407] | | 263 [183-301] | 198 [165-251] | **0.013** |
| Lymphocytes (cells/µL) | 1444 [1206-1638] | | 1735 [1478-2414] | 1828 [1303-2076] | **0.034** |

Continuous variables are expressed as median [IQR]. Comparisons between continuous variables were made by using the non-parametric Kruskal-Wallis test. *p* values <.05 were considered statistically significant. TfSI: transferrin saturation index; sTfR: soluble transferrin receptor; hsCRP: high sensitivity C-reactive protein; RTL: relative telomere length.
