# Supplementary material for: Higher plasma levels of thymosin-α1 are associated with a lower waning of humoral response after COVID-19 vaccination: an eight months follow-up study in a nursing home
Source: Immun Ageing. 2023 Mar 6;20:9. doi: 10.1186/s12979-023-00334-y (PMC9986663; doi:10.1186/s12979-023-00334-y)
Supplement: Supplementary file 5 — Additional file 5: Supplementary Fig. 2. Levels of Thymosin-α1 in higher vs. lower responders of the older group. [file 12979_2023_334_MOESM5_ESM.docx]

**ADDITIONAL INFORMATION 5.**

**SUPPLEMENTARY FIGURE 2. LEVELS OF THYMOSIN-A1 IN HIGHER VS. LOWER RESPONDERS OF THE OLDER GROUP AND YOUNGER PARTICIPANTS**

Comparison of thymosin-α1 levels between higher (≥1410 BAU/mL) and lower (<1410 BAU/mL) responders at T1 among the older group. Attending to the mixed phenotype of older people regarding the T1 humoral response represented in Figure 1A, we chose the minimum value of anti-S IgG titers of the upper subgroup as the threshold to segregate both subgroups. Higher responders had higher levels of thymosin-α1 than lower responders but a similar level than that of the younger group. Additionally, higher responders showed also higher levels of CD3 (*p*=0.024), CD4 (*p*=0.038) and lymphocytes (*p*=0.02) counts, as well as higher levels of total IgG (*p*=0.006) and calcium (*p*=0.017), compared with older participants with lower responses (not shown).
