# Supplementary material for: Higher plasma levels of thymosin-α1 are associated with a lower waning of humoral response after COVID-19 vaccination: an eight months follow-up study in a nursing home
Source: Immun Ageing. 2023 Mar 6;20:9. doi: 10.1186/s12979-023-00334-y (PMC9986663; doi:10.1186/s12979-023-00334-y)
Supplement: Supplementary file 6 — Additional file 6: Supplementary Fig. 3. Correlations among Log of anti-S antibody titers at all different study points. [file 12979_2023_334_MOESM6_ESM.docx]

**ADDITIONAL INFORMATION 6.**

## Supplementary Figure 3. Correlations AMONG log of anti-S antibody titers at all different study points.

S1A

S1D


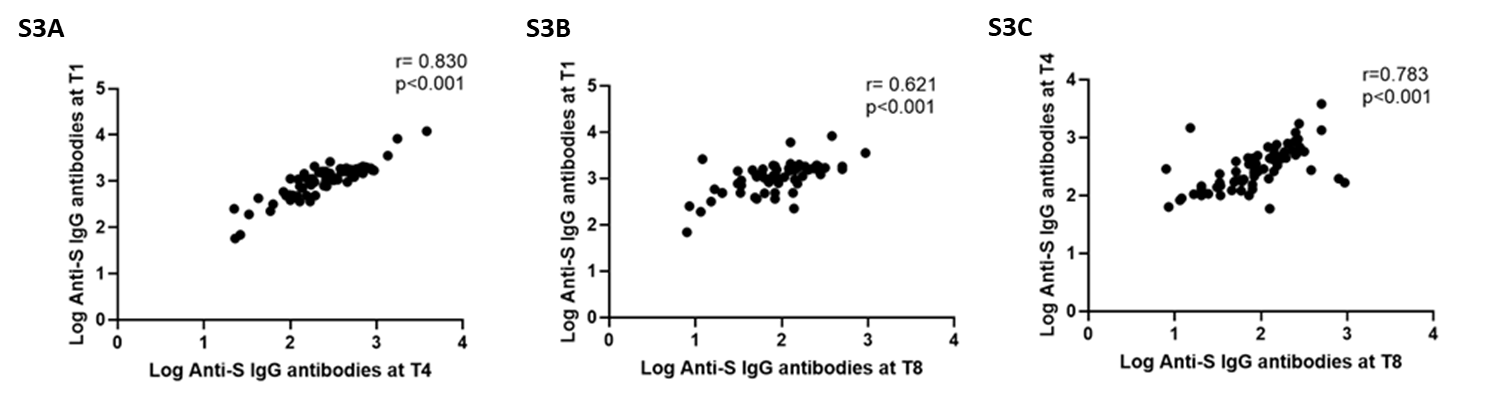
Spearman’s correlation analysis between log of anti-S antibody titers at T1 and T4 (S3A), between log of anti-S antibody titers at T1 and T8 (S3B) and between log of anti-S antibody titers at T4 and T8 (S3C). *p*<0.05 was considered statistically significant.
