# Supplementary material for: Higher plasma levels of thymosin-α1 are associated with a lower waning of humoral response after COVID-19 vaccination: an eight months follow-up study in a nursing home
Source: Immun Ageing. 2023 Mar 6;20:9. doi: 10.1186/s12979-023-00334-y (PMC9986663; doi:10.1186/s12979-023-00334-y)
Supplement: Supplementary file 7 — Additional file 7: Supplementary Fig. 4. Factors associated with the magnitude of the initial response to the BNT162B2 vaccine at T1 by age-groups. [file 12979_2023_334_MOESM7_ESM.docx]

**ADDITIONAL INFORMATION 7**

## **SUPPLEMENTARY FIGURE 4.** **FACTORS ASSOCIATED WITH THE MAGNITUDE OF THE INITIAL RESPONSE TO THE BNT162B2 VACCINE AT T1 BY AGE-GROUPS.**

Spearman’s correlation analysis between potential age-related factors associated to the magnitude of the initial response to the vaccine (T1, one month after the second dose of the vaccine) in the entire population, as well as in age-stratified groups (young, middle-age and older). Color intensity of boxes represents Spearman's rank correlation coefficient value as indicated in the color legend. All colored boxes represent statistically significant correlations (*p* values <0.05). * *p* values between 0.1 and 0.05
