# Supplementary material for: Higher plasma levels of thymosin-α1 are associated with a lower waning of humoral response after COVID-19 vaccination: an eight months follow-up study in a nursing home
Source: Immun Ageing. 2023 Mar 6;20:9. doi: 10.1186/s12979-023-00334-y (PMC9986663; doi:10.1186/s12979-023-00334-y)
Supplement: Supplementary file 8 — Additional file 8. Detailed methodology. [file 12979_2023_334_MOESM8_ESM.docx]

**ADDITIONAL INFORMATION 8**

**DETAILED METHOLOGY**

**Immune subsets**

Absolute numbers of total lymphocytes, as well as CD3, CD4, and CD8 T-cells, B-cells, and NK-cells counts, were routinely determined in fresh blood samples collected at T1 at the Immunology Service of our hospital, following standard procedures. Identification of total CD3+, CD3+CD4+, CD3+CD8+, CD3+CD4+/CD3+CD8+ lymphocyte counts were performed on peripheral whole blood samples stained with AQUIOS Tetra-1 Panel Monoclonal Antibody Reagents, a four-color monoclonal antibody cocktail consisting of CD45-FITC/CD4-RD1/CD8-ECD/CD3-PC5. In a separate tube, whole blood samples were stained with AQUIOS Tetra-2+ Panel Monoclonal Antibody Reagents, a four-color monoclonal antibody cocktail consisting of CD45-FITC/(CD56 + CD16)-RD1/CD19-ECD/CD3-PC5, and used for the identification of total CD3-CD19+, CD3-CD56+ and/or CD16+ lymphocytes. Flow cytometric analyses was performed on a Navios EX flow cytometer (Beckman-Coulter, California) and the different cell populations were analyzed using the Navios EX Software v2.0. Reference values for all immune cellular subsets analyzed are provided in the table below.

| **Immune Cell Subsets** | **Reference Values** |
| --- | --- |
| CD3 (cells/µL) | 700-2100 |
| CD4 (cells/µL) | 300-1400 |
| CD8 (cells/µL) | 200-900 |
| B cells (cells/µL) | 100-500 |
| Nk cells (cells/µL) | 90-600 |
| Lymphocytes (cells/µL) | 1000-2800 |

**Biochemical soluble biomarkers.**

Biochemical soluble markers, including blood proteins, micronutrients, soluble iron metabolism-related markers and inflammatory biomarkers, were routinely determined by standard procedures at the Biochemistry Service of our hospital. Blood proteins, micronutrients, transferrin and soluble transferrin receptor (sTfR) were measured by photometry and ferritin by particle enhanced immunoturbidimetric assay in a Hitachi Cobas C702 modular analyzer (Roche Diagnostics, Rotkreuz, Switzerland). TfSI was estimated as (total plasma iron (µg/dL) x100)/(transferrin (mg/dL)x1.27). hsCRP and β2-microglobulin levels were determined in frozen serum samples with an immunoturbidimetric assay using Cobas 701 (Roche Diagnostics, Mannheim, Germany). Measurements of the levels of homocysteine were performed by photometry according to manufacturer’s instructions, and D-dimer levels were quantified by using an automated latex enhanced immunoassay using frozen plasma samples (HemosIL D-Dimer HS 500, Instrumentation Laboratory, Bedford, Massachusetts). Reference values for biochemical soluble markers are given in the table below.

| **Biochemical Soluble Markers** | **Reference Values** |
| --- | --- |
| Albumin (g/dL) | 3.5-5.0 |
| IgA (mg/dL) | 40-350 |
| IgM (mg/dL) | 50-300 |
| IgG (mg/dL) | 700-1600 |
| Vitamin B12 (pg/mL) | 191-663 |
| Folate (ng/mL) | 2.9-16.9 |
| Calcium (mg/dL) | 8.5-10.5 |
| Phosphorous (mg/dL) | 2.7-4.5 |
| Sodium (mEq/L) | 135-145 |
| Potassium (mEq/L) | 3.5-5.1 |
| Magnesium (mg/dL) | 1.80-2.60 |
| Iron (µg/dL) | 65-175 |
| TfSI (%) | 15-45 |
| Transferrin (mg/dL) | 215-365 |
| sTfR (mg/L) | 2.2-5.0 |
| Ferritin (ng/mL) Men | 30-400 |
| Homocysteine (mg/L) | 0.0-2.0 |
| β-2 microglobulin (mg/L) | 1.50-2.50 |
| hsCRP (mg/L) | 0.0-3.0 |
| D-Dimers (ng/mL) | 1-500 |

**RTL quantification**

For RTL quantification, copy number quantifications were performed by quantitative polymerase chain reaction (qPCR). Copy number quantifications were performed by qPCR following a standard protocol [*]. We used 60 ng of DNA for each reaction and determined the ratio between number of copies of the telomere sequence and the single copy gen Beta-globin. Primers sequences (5´-3´) were: Telomere Forward (GGTTTTTGAGGGTGAGGGTGAGGGTGAGGGTGAGGGT) and Reverse (TCCCGACTATCCCTATCCCTATCCCTATCCCTATCCCTA). For each reaction, 60 ng of DNA were used.; Human Beta-Globin Forward (ACACAACTGTGTTCACTAGG) and Reverse (CAACTTCATCCACGTTCACC). The fluorescent reading for the copy number quantification was performed in a Light-cycler 480 (Roche). (Extended in Supplementary Methodology).

* Cawthon RM. Telomere length measurement by a novel monochrome multiplex quantitative PCR method. Nucleic Acids Res. 2009; 37:e21.

### ***Thymic output quantification (sj/β-TRECs ratio)***

The thymic output was calculated as the sj/β-TRECs ratio by Droplet Digital PCR (ddPCR), in a single reaction, optimized from Profaizer T et al., 2020 [1] and according to the manufacturer’s recommendations, in a QX200 system (BIORAD). Primers and probes design were optimized from Ferrando-Martinez et al., 2010 [2]. Each ddPCR reaction containing 150 ng of DNA extracted from cryopreserved PBMC, 1x ddPCR Supermix no UTP for Probes (BIORAD), 250 nM HEX labelled Delta Probe, 250 nM of FAM labelled Beta Probe, 1 µM of 6 different Beta Forward primers, 1 µM of Beta reverse primer, 1 µM of Delta forward primer and 1 µM of Delta reverse primers. The final volume of the reaction was 20 µl. The results were analyzed by using the Quantasoft 1.7.1 Software. The limit of detection was 1 copy by reaction.

1. Profaizer T, Slev P. A multiplex, droplet digital PCR assay for the detection of T-cell receptor excision circles and kappa-deleting recombination excision circles. Clin Chem. 2020; 66:229–38.

2. Ferrando-Martínez S, Franco JM, Ruiz-Mateos E, et al. A reliable and simplified sj/β-TREC ratio quantification method for human thymic output measurement. J Immunol Methods. 2010; 352:111–7.

### ***Thymosin-α1 quantification***

Thymosin-α1 was quantified in plasma samples using the Human Thymosin-α1 competitive ELISA kit (MyBiosurce®) for research purposes only, following manufacturer’s instructions. Absorbance for thymosin-α1 were read at 450nm using a CLARIOstar® microplate reader (BMG labtech, Ortenberg, Germany). The limit of detection was 1 ng/mL.
